# Exploration of the Key Proteins of High-Grade Intraepithelial Neoplasia to Adenocarcinoma Sequence Using In-Depth Quantitative Proteomics Analysis

**DOI:** 10.1155/2021/5538756

**Published:** 2021-11-29

**Authors:** Yin Zhang, Chun-Yuan Li, Meng Pan, Jing-Ying Li, Wei Ge, Lai Xu, Yi Xiao

**Affiliations:** ^1^Division of Colorectal Surgery, Department of General Surgery, Peking Union Medical College Hospital, Chinese Academy of Medical Sciences and Peking Union Medical College, Beijing, China; ^2^State Key Laboratory of Medical Molecular Biology & Department of Immunology, Institute of Basic Medical Sciences Chinese Academy of Medical Sciences, School of Basic Medicine Peking Union Medical College, Beijing, China; ^3^Hebei Key Laboratory of Chronic Kidney Diseases and Bone Metabolism, Affiliated Hospital of Hebei University, Baoding, China

## Abstract

**Purpose:**

In this study, we aimed to provide a comprehensive description of typical features and identify key proteins associated with the high-grade intraepithelial neoplasia- (HIN-) adenocarcinoma (AC) sequence.

**Methods:**

We conducted tandem mass tag-based quantitative proteomic profiling of normal mucosa, HIN, and AC tissues. Protein clusters representative of the HIN-AC sequence were identified using heatmaps based on Pearson's correlation analysis. Gene ontology (GO), Kyoto Encyclopedia of Genes and Genomes (KEGG), and Reactome analyses were performed using the Database for Annotation, Visualization, and Integrated Discovery (DAVID) database, ClueGO plugin in Cytoscape, and the Metascape database. The prognostic value of the key proteins and their effects on the tumor microenvironment and consensus molecular subtype were explored based on The Cancer Genome Atlas.

**Results:**

We identified 536 proteins categorized into three clusters. Among the biological processes and pathways of the highly expressed proteins in the HIN-AC sequence, proteins were predominantly enriched in response to gut microbiota, cell proliferation, leukocyte migration, and extracellular matrix (ECM) organization events. SERPINH1 and P3H1 were identified as the key proteins that promote the HIN-AC sequence. In the correlation analysis of infiltrating immune cells, both SERPINH1 and P3H1 expression correlated negatively with tumor purity, while correlating positively with abundance of CD8^+^ T cells, B cells, macrophage/monocytes, dendritic cells, cancer-associated fibroblasts, endothelial cells, neutrophils, and natural killer cells. Furthermore, both SERPINH1 and P3H1 expression positively correlated with common immune checkpoints and mesenchymal molecular subtype. High P3H1 expression was associated with poor disease-free survival and overall survival.

**Conclusions:**

ECM-related biological processes and pathways are typical features of the HIN-AC sequence. SERPINH1 and P3H1 might be the key proteins in this sequence and be related to ECM remodeling and immune suppression status in CRC.

## 1. Introduction

Colorectal cancer (CRC) is the second leading cause of all cancer deaths, accounting for 9.2% worldwide [1]. In the most common etiology of CRC, the conventional adenoma to carcinoma sequence accounts for approximately 85% of cases [2]. High-grade intraepithelial neoplasia (HIN), characterized by cribriform architecture and/or severe cytologic atypia, is an advanced form of adenoma (tumor size >1 cm, villous/tubulovillous adenoma, or/and HIN) with a high risk of carcinogenesis [3]. HIN is associated with a higher risk of progression to CRC than tubular adenoma after removal of polyps (63 of 2,048 versus 171 of 12,786) [4]. The histology of HIN is very similar to that of cancer and is confined in the epithelial layer with almost no risk of metastasis. Moreover, many cases of HIN diagnosed through biopsy have been identified as invasive colorectal cancer through analysis of the surgical specimens [5].

Many studies have focused on the markers that show diagnostic value or have been identified as therapeutic targets in the normal adenoma-carcinoma sequence. Zhang et al. found that mTOR, p70s6 K, and 4EBP1 were highly expressed in HIN and CRC compared with normal mucosa (NM), and mTOR gene silencing was implicated as a novel therapeutic strategy for CRC [6]. Dipeptidase 1 (DPEP1) was upregulated in HIN and CRC compared with low-grade intraepithelial neoplasia and NM [7]. Furthermore, high DPEP1 expression is strongly associated with poor prognosis in CRC patients, indicating that this protein plays an important role in carcinogenesis and might contribute to cancer development [7]. Similarly, other studies have investigated proteins that could be both early diagnostic markers in adenoma carcinogenesis and prognostic markers in CRC [8–10]. However, these studies did not focus on the HIN-AC sequence in carcinogenesis. The HIN-AC sequence is the advanced phase of carcinogenesis and the proteins or/and pathways involved might be both preventive and therapeutic target. Hence, elucidation of the events that promote the HIN-AC sequence is crucial for effective management of CRC. In this study, we aimed to provide a comprehensive description of the key proteins involved in the HIN-AC sequence.

## 2. Methods

### 2.1. Patients and Tissue Samples

NM, HIN, and AC tissues were obtained from 24 patients recruited at the Division of Colorectal Surgery, Department of General Surgery, Peking Union Medical College Hospital (China). Details of the patients and tissues are listed in Table S1. After removal, all tissues were stored temporarily on dry ice and then transferred to −80°C.

This study was approved by the Ethics Committee of Peking Union Medical College Hospital (Number: JS-2094). Written informed consent was obtained from each patient prior to study commencement.

### 2.2. Protein Extraction and Tandem Mass Tag-Labeling

Frozen HIN, AC, and NM tissues were homogenized with lysis buffer mixed with 8 M urea in phosphate-buffered saline (PBS), 1 × protease inhibitor cocktail, and 1 mM phenylmethylsulfonyl fluoride (PMSF). Proteins were acquired by centrifugation of the tissue homogenate (12,000 rpm for 15 min at 4°C) and the protein concentration was measured using a Nanodrop 2000 (Thermo Fisher Scientific, Waltham, MA, USA). The proteins in each group were the alkylated with dithiothreitol (DTT) and iodoacetamide (IAA). Protein digestion was performed using trypsin/Lys-C mix at a protein/protease ratio of 25 : 1. TMT isobaric label reagents were used to label each group as follows: NM group, TMT-129; HIN group, TMT-126; and AC group, TMT-130. The TMT-labeled peptides were then analyzed by high performance liquid chromatography (HPLC) and LC-MS/MS according to previously described methods [11].

### 2.3. Protein Identification

Proteins were identified with Proteome Discoverer 2.2 software (Thermo Fisher Scientific) and the SEQUEST search engine using the reviewed Swiss-Prot human FASTA database of UniProt as the reference. Proteins with a false discovery rate (FDR) < 0.01 and unique peptides ≥2 qualified for further analysis. Proteins were quantified using the TMT-6plex method. The mass spectrometry proteomics data have been deposited with the ProteomeXchange Consortium (https://proteomecentral.proteomexchange.org) in the iProX partner repository with the dataset identifier: PXD023899 [12].

### 2.4. Bioinformatics Analysis

Heatmaps of differentially expressed proteins were generated using HCE 2.3 software based on the filtered proteomic profiles of NM, HIN, and AC tissues based on a fold change (FC) in expression >1.3 between HIN and AC. Gene ontology (GO) categories including GO-biological process (BP), GO-cellular component (CC), and GO-molecular function (MF) were analyzed using the Database for Annotation, Visualization, and Integrated Discovery (DAVID) [13]. Kyoto Encyclopedia of Genes and Genomes (KEGG) and Reactome pathway analyses were performed using the ClueGO and CluePedia plugins in Cytoscape [14–16]. Protein-protein interaction (PPI) networks were constructed using Search Tool for Retrieval of Interacting Genes/Proteins (STRING) database [17]. The core clusters were identified using the MCODE plugin and the key proteins were identified using the CytoHubba plugin in Cytoscape [18, 19]. The BPs and pathways of core clusters were identified and downloaded in Metascape [20]. The microarray data of GSE 41657 and GSE 37364 were downloaded from the Gene Expression Omnibus (GEO) database. Survival was evaluated by Gene Expression Profiling Interactive Analysis (GEPIA) based on the gene expression data of CRC in the Cancer Genome Atlas (TCGA) [21]. Immune cell infiltration was estimated using the Tumor Immune Estimation Resource (TIMER) database [22].

### 2.5. Statistical Analysis

Statistical analysis was performed using GraphPad Prism 8.0.1 (GraphPad Software, Inc., La Jolla, CA, USA). Student's *t*-test or analysis of variance (ANOVA) was used to evaluate quantitative data. Pearson's correlation analysis was performed to evaluate associations between sets of data, and Spearman's correlation analysis was performed to evaluate associations between gene expression and abundance of infiltrating immune cells. The Kaplan–Meier method was used for survival analysis. *P* < 0.05 was considered to indicate statistical significance.

## 3. Results

### 3.1. Enrichment Analysis of the Proteins Promoting HIN Carcinogenesis

**Figure 1:**
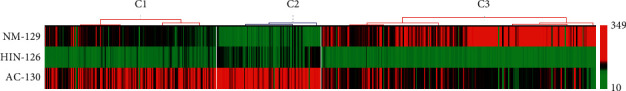
Heatmap of the highly expressed proteins in HIN-AC sequence. Heatmap showing the relative abundance of 536 proteins in the normal mucosa (NM), high-grade intraepithelial neoplasia (HIN), and adenocarcinoma (AC) group. The proteins were clustered hierarchically by Pearson correlation analysis. Green indicates downregulated proteins, whereas red indicates upregulated proteins. C: cluster.

**Figure 2:**
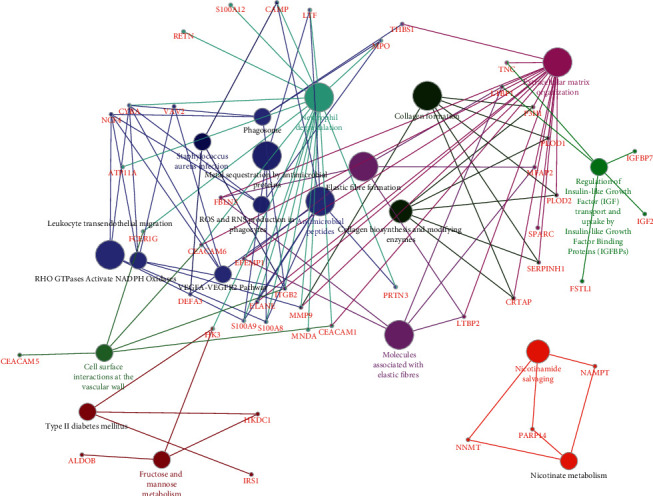
Pathway enrichment analysis of the proteins increasingly expressed in NM-HIN-AC sequence. Kyoto encyclopedia of gene (KEGG) and Reactome pathway analyses of cluster 2 were performed using the ClueGO and CluePedia plugins in Cytoscape. Circles shown in the same color represent similar enrichment results.

The clinical characteristics of the NM, HIN, and AC tissue samples obtained from patients are listed in Table S1. The workflow of this study is shown in Figure S1. We identified a total of 5,665 proteins according to the criteria described in the Methods section. Based on the criterion of FC > 1.3 between HIN and AC, we selected the 536 upregulated proteins for clustering analysis (Figure 1). The proteins in cluster 2 showed the increasing trend of the NM-HIN-AC sequence. Therefore, we focused on the proteins in cluster 2 (102 proteins) in the subsequent enrichment analysis. In the GO-CC analysis, we found most of the proteins were located in the extracellular region (Table 1). In the GO-MF analysis, “calcium ion binding” was enriched significantly (Table 2). After synthesizing the top 20 categories of the GO-BP analysis, “response to gut microbiota,” “cell proliferation,” “leukocyte migration,” and “extracellular matrix (ECM) organization” were identified as representative events in the HIN-AC sequence (Table 3). In the KEGG and Reactome pathway analyses, the upregulated proteins were enriched in extracellular matrix organization, collagen formation, molecules associated with elastic fibers, neutrophil degranulation, antimicrobial peptides, *Staphylococcus aureus* infection, and cell surface interaction (Figure 2).

### 3.2. Identification of the Core Clusters in the HIN-AC Sequence

**Figure 3:**
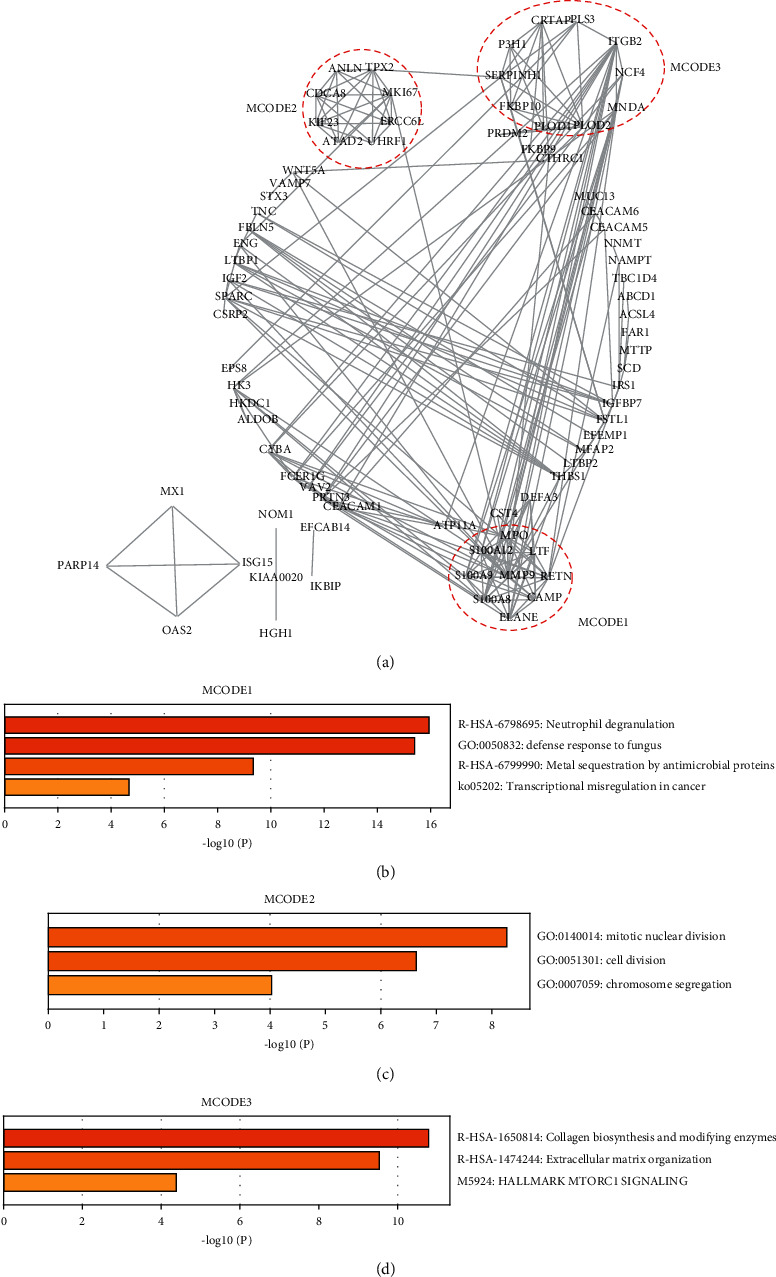
Identification of the core clusters of the proteins upregulated in the NM-HIN-AC sequence. (a) Protein-protein interaction (PPI) network of the proteins upregulated in the NM-HIN-AC sequence was constructed using STRING. The top 3 clusters were identified using the MCODE plugin in Cytoscape. (b–d) Enrichment analysis of MCODE1-MCODE3 using Metascape.

**Figure 4:**
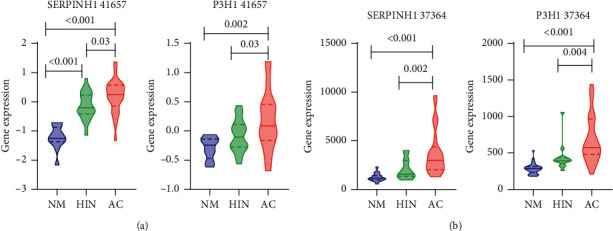
Validation of the expression of SERPINH1 and P3H1 in the GEO database. (a) The expression of SERPINH1 and P3H1 was significantly upregulated in the HIN-AC sequence in the GSE 41657 dataset. (b) The expression of SERPINH1 and P3H1 was significantly upregulated in the HIN-AC sequence in the GSE 37364 dataset.

We then constructed the PPI network and identified the core clusters using the MCODE plugin in Cytoscape (Figure 3(a)). We selected the top three MCODE clusters for enrichment analysis (Figures 3(b)–3(d)). Neutrophil degranulation, defense response to fungus, and metal sequestration by antimicrobial proteins were enriched in MCODE1 (Figure 3(b)). Mitotic nuclear division, cell division, and chromosome segregation were enriched in MCODE2 (Figure 3(c)). Collagen biosynthesis and modifying enzymes and extracellular matrix organization were enriched in MCODE3 (Figure 3(d)). In these three MCODE clusters, MCODE2 was associated with cell division and reflected the hallmark characteristic of cancer cells. The interaction of MCODE 1 and MCODE 3 was abundant and five proteins (ELANE, S100A8, S100A9, S100A12, and MMP9) in MCODE 1 were matrisome-associated proteins [23]. Moreover, the interaction between cancer cells and ECM components is a crucial event that promotes tumor invasion and metastasis. Hence, we focused on MCODE3 in the next step of our analysis. The CytoHubba plugin was used to screen the hub proteins in the MCODE3. SERPINH1 and P3H1 were identified as the intersection proteins using the maximal clique centrality (MCC), density of maximum neighborhood component (DMNC), maximum neighborhood component (MNC), and clustering coefficient methods in CytoHubba (Table S2). Thus, we regarded SERPINH1 and P3H1 as key proteins in the HIN to AC process. We then selected the GSE 41657 and GSE 37364 datasets from the GEO database to validate the expression of SERPINH1 and P3H1 in NM, HIN, and AC tissues. In the two datasets, SERPINH1 and P3H1 were both significantly upregulated between HIN and AC tissues (Figures 4(a) and 4(b)).

### 3.3. SERPINH1 and P3H1 Expression Correlates with the Immune Infiltration in CRC

**Figure 5:**
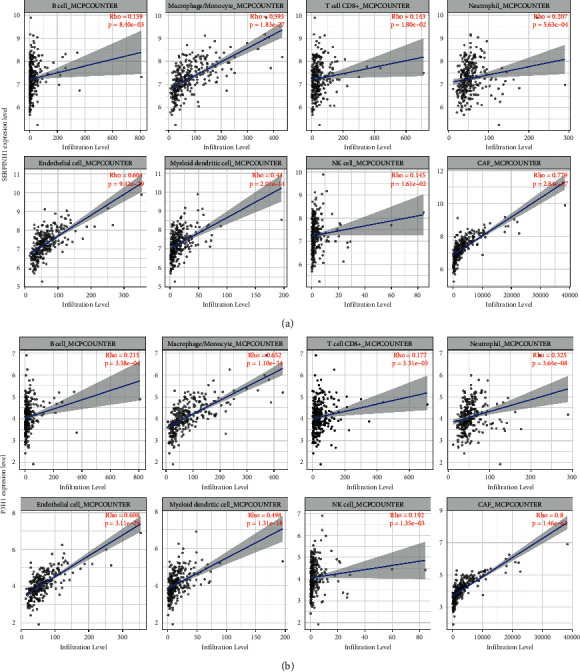
Correlation analysis of the expression of SERPINH1 and P3H1 and the abundance of immune infiltrating cells. (a) Spearman's correlation analysis of SERPINH1 expression and the abundance of immune infiltrating cells using the MCP-counter algorithm. (b) Spearman's correlation analysis of P3H1 expression and the abundance of immune infiltrating cells using the MCP-counter algorithm. NK cell, natural killer cell; CAF, cancer associated fibroblasts.

**Figure 6:**
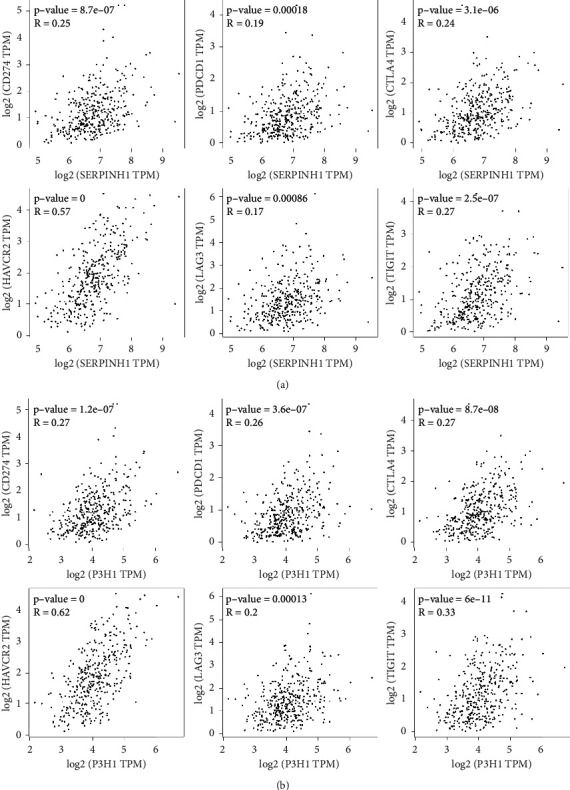
Correlation analysis of the expression of SERPINH1 and P3H1 and the expression of common CRC immune checkpoints. (a) Pearson correlation analysis of the expression of SERPINH1 and PDCD1, CD274, CTLA4, HAVCR2, LAG3, and TIGIT in the GEPIA database. (b) Pearson correlation analysis of the expression of P3H1 and PDCD1, CD274, CTLA4, HAVCR2, LAG3, and TIGIT in the GEPIA database.

**Figure 7:**
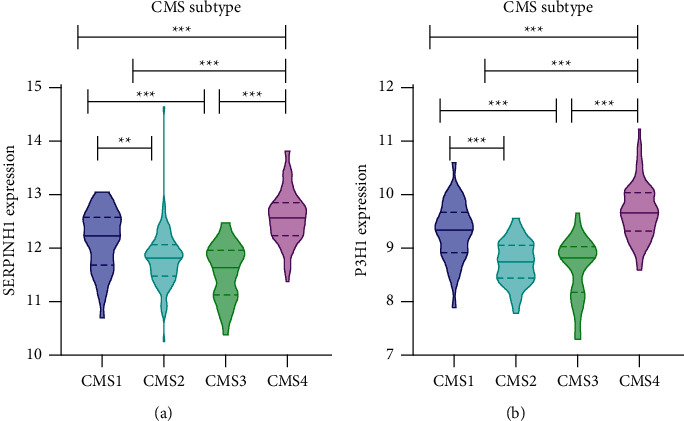
Expression of SERPINH1 and P3H1 in different consensus molecular subtypes (CMS) of CRC. The expression of SERPINH1 (a) and P3H1 (b) was significantly upregulated in CMS4 of CRC in TCGA cohort.

After identification and validation of the expression of SERPINH1 and P3H1 in the HIN carcinogenesis process, we further explored the potential correlation of these two key proteins with the immune infiltration of CRC. SERPINH1 and P3H1 both correlated negatively with tumor purity and positively with CD8^+^ T cells, B cells, macrophages/monocytes, dendritic cells (DC), cancer associated fibroblasts (CAF), endothelial cells, neutrophils, and natural killer (NK) cells (Figures 5(a) and 5(b)). This result was in accordance with the enrichment in “leukocyte migration” in the GO-BP analysis of the HIN-AC sequence and indicated that SERPINH1 and P3H1 are continuously expressed during HIN carcinogenesis. The recruitment of CAF and endothelial cells was associated with cancer progression. Although the high expression of SERPINH1 and P3H1 was related to the high abundance of immune cell infiltration, we analyzed the correlation of these proteins with common immune checkpoints of CRC in the GEPIA database to determine the potential pro- or anticytotoxic effects of the inflammatory microenvironment on cancer cells [24]. The expression of both SERPINH1 and P3H1 correlated positively with the expression of PD-1 (PDCD1), PD-L1 (CD274), TIGIT, LAG3, TIM3 (HAVCR2), and CTLA4 (Figures 6(a) and 6(b)). Next, we evaluated the expression of SERPINH1 and P3H1 in the four consensus molecular subtypes (CMS) in TCGA database. The two proteins were significantly upregulated in CMS4 compared with the other three subtypes, which indicated that SERPINH1 and P3H1 correlate with the mesenchymal phenotype (Figure 7). SERPINH1 has been identified as a CRC risk factor in previous studies [25–27]. Hence, we selected P3H1 for further analysis and found that the high expression of P3H1 was significantly associated with poor prognosis of CRC patients in TCGA datasets (Figure S2). Thus, P3H1 was implicated as a potential prognostic biomarker in CRC patients.

## 4. Discussion

In this study, we performed a quantitative proteomics analysis of NM, HIN, and AC tissues and focused on the proteins upregulated from HIN to AC in order to identify the pivotal events and proteins that might promote the HIN-AC sequence. SERPINH1 and P3H1 were identified as key proteins in ECM organization and collagen formation, which might play a core role in HIN carcinogenesis. Furthermore, our analysis of infiltrating cells and immune checkpoints indicated that SERPINH1 and P3H1 are associated with immune escape. In the CMS analysis, SERPINH1 and P3H1 expression were significantly associated with CMS4 (mesenchymal type), indicating that SERPINH1 and P3H1 are related to ECM remodeling events. Our analysis also implicated P3H1 as a potential prognostic biomarker in CRC.

HIN is a type of advanced adenoma with a high risk of progression to AC (rate ratio [RR]: 2.7; 95% confident incidence [CI]:1.9–3.7) [28]. A comprehensive understanding of the processes and the pathways involved in the HIN-AC sequence will provide a reference for the development of strategies for its prevention. There is high-quality evidence showing the effectiveness of aspirin as the primary strategy for CRC chemoprevention [29, 30]. The Aspirin Folate Polyp Prevention Study showed that low-dose aspirin decreased the risk of adenoma (relative risk, 0.81; 95% CI, 0.69–0.96) and advanced adenoma/carcinoma (relative risk, 0.59; 95% CI, 0.38–0.92) [31]. However, in the Colorectal Adenoma/Carcinoma Prevention Programme 1 (CAPP1) study, aspirin did not decrease the colonic polyp burden in familial adenomatous polyposis (FAP) [32]. Moreover, the specific mechanism underlying the role of aspirin in this process remains to be fully clarified. To fulfill the requirements of precision medicine, future methods of CRC chemoprevention should be focused on the targetable tumorigenesis pathways [30]. Furthermore, from the perspective of tumorigenesis, HIN is the advanced stage of the adenoma-carcinoma sequence. Thus, it can be hypothesized that the critical malignancy-promoting events occur in the HIN-AC process, with key proteins in this process acting as the “trigger” of the invasive and metastatic abilities that promote cancer development. Hence, these key proteins might be targets for the prevention of CRC.

In our analysis, we found that “ECM organization,” “collagen formation,” “defense response to bacteria and fungus,” “neutrophil degranulation,” and “cell proliferation” were the core events in the HIN-AC sequence. Sustained proliferative signaling is the canonical hallmark of cancer [33]. There are three major types of cytoplasmic granules: azurophilic granules (primary granules), specific granules (secondary granules), and gelatinase granules (tertiary granules) [34]. Azurophilic granules, which contain myeloperoxidase (MPO) as well as numerous proteolytic and bactericidal proteins, function as a microbicidal compartment that is mobilized during phagocytosis [34]. Specific granules interact with gelatinase granules to remodel the ECM [35]. Proteogenomic research has shown that neutrophil degranulation is associated with an immunosuppressive phenotype in lung adenocarcinoma and promotes lymph node metastasis in breast cancer [36]. It can be speculated that neutrophil degranulation is the reflection of a local inflammatory reaction caused by variation in the gut microbiota. *Fusobacterium nucleatum*, *Escherichia coli*, and enterotoxigenic *Bacteroides fragilis* have been implicated in colorectal carcinogenesis [37]. Yang et al. found that the abundance of *Eubacterium*, *Roseburia*, *Faecalibacterium*, and *Oscillospira* decreased significantly from advanced adenoma to CRC [38]. Some studies have indicated that ECM-related events play a role in the adenoma-carcinoma sequence, which is consistent with our findings. Fonseca et al. reported that the extracellular remodeling process was significantly enriched during adenoma-carcinoma progression [39]. Using bioinformatics analysis and experimental validation, Hauptman et al. identified six ECM-related proteins (DCN, EPHA4, FN1, SPARC, SPON2, and SPP1) that play an important role in colorectal carcinogenesis [40, 41]. Versican, a large extracellular matrix proteoglycan that regulates many malignant biological processes, was highly expressed in the stroma of high-risk adenomas and carcinomas compared with low-risk adenomas [42]. In our analysis, collagen formation was identified as the representative pathway of the ECM organization. Birk et al. found higher collagen intensity and more aligned collagen deposition in aligned colon cancer compared with HIN [43]. Furthermore, second-harmonic generation imaging indicated enhanced collagen formation in the HIN-AC sequence. On the basis of these studies and our own analyses, we speculated that some BPs and pathways promote not only CRC development, but also HIN carcinogenesis.

SERPINH1 and P3H1 play pivotal roles in collagen maturation. SERPINH1 is a collagen-specific chaperone that is localized in the endoplasmic reticulum. This protein prevents local unfolding and/or aggregation of procollagen and promotes collagen I synthesis and secretion [25]. SERPINH1 is associated with ulcerative colitis-associated carcinomas, local lymph node metastasis, chemotherapy resistance, and poor prognosis in CRC [26, 27, 44]. SERPINH1, and its dependent collagen secretion might promote cancer metastasis through cancer cell-platelet interactions [45]. P3H1 catalyzes the posttranslational formation of 3-hydroxyproline at -Xaa-Pro-Gly- sequences in collagens, especially types IV and V. P3H1 was identified as a risk factor for hepatocellular carcinoma by bioinformatics analysis, which also indicated that this protein activates the PI3K/AKT signaling pathway to promote the development of osteosarcoma [46, 47]. In our analysis, high expressions of SERPINH1 and P3H1 were found to correlate positively with immune infiltration and immune checkpoints in the tumor microenvironment. We speculated that SERPINH1 and P3H1 represent potential targets that might act synergistically to enhance the effect of immune checkpoint inhibitors (ICIs). Furthermore, SERPINH1 and P3H1 were highly expressed in the CMS4 of CRC. CMS of CRC was performed based on gene expression and classified into four subtypes (CMS1, microsatellite instability immune; CMS2, canonical; CMS3, metabolic; and CMS4, mesenchymal) [48]. The typical molecular characters of CMS4 are epithelial-mesenchymal transition, ECM remodeling, CAF infiltration, and TGF-*β* activation [48]. The typical clinicopathological characters of CMS4 are stroma infiltration and poor prognosis [48–50]. Therefore, SERPINH1 and P3H1 might remodel the ECM and establish a local immunosuppressive environment at the stage of HIN-AC and continue promoting the development of CRC.

Some limitations of this study should be noted. First, this study was conducted using TMT-labeling of the mixed tissues rather than individual tissues in each of the study group. Only a small amount of HIN tissue is obtained, and the majority is used for pathological diagnosis. Thus, the tissues available for our research were too limited to perform individual proteomic experiments. We mixed tissues after strict quantitation in order to guarantee the concentration of the protein in the sample and the accuracy of quantitative results in each group. Compared with a label-free approach, it is not possible to avoid batch effects when using the labeling approach with mixed samples. However, the TMT-labeling approach (6-plex) is sensitive and has the capacity to identify more proteins than the iTRAQ and label-free approaches [51]. Second, the results are based mainly on proteomic analysis without experimental validation; therefore, the specific roles of the core BPs and pathways that promote the HIN-AC sequence remain to be elucidated. An in-depth exploration of the mechanism by which SERPINH1 and P3H1 promote the transition from HIN to AC is also warranted. Third, the prognostic value of P3H1 requires confirmation in a large cohort. Fourth, our enrichment analysis of the HIN-AC sequence indicates that the variation of gut microbiota promotes the transition. Therefore, a combination of metagenomics and metabolomics approaches may provide a more comprehensive understanding the HIN-AC sequence.

## 5. Conclusion

We comprehensively analyzed the proteomic profiles of NM, HIN, and AC. “ECM organization,” “collagen formation,” “defense response to bacteria and fungus,” “neutrophil degranulation,” and “cell proliferation” were identified as the core events of the HIN-AC sequence. SERPINH1 and P3H1 might be the key proteins in this sequence. Furthermore, our findings indicate that SERPINH1 and P3H1 are related to ECM remodeling and immune suppression status in CRC. P3H1 is a potential risk factor of CRC.

## Figures and Tables

**Table 1:** GO-CC analysis of HIN-AC sequence.

GO-CC term	Count	*P* value
Extracellular space	31	1.13*E* − 09
Extracellular region part	51	6.25*E* − 09
Extracellular region	54	1.04*E* − 07
Extracellular exosome	40	1.59*E* − 07
Extracellular vesicle	40	1.82*E* − 07
Extracellular organelle	40	1.84*E* − 07
Membrane-bounded vesicle	45	6.84*E* − 07
Extracellular matrix	16	8.13*E* − 07
Secretory granule	11	7.24*E* − 05
Proteinaceous extracellular matrix	11	7.76*E* − 05
Cytoplasmic vesicle part	13	3.97*E* − 04
Secretory vesicle	11	6.14*E* − 04
Endoplasmic reticulum part	18	8.02*E* − 04
Cytoplasmic, membrane-bounded vesicle	17	0.002303304
Endoplasmic reticulum	21	0.0024862
Cytoplasmic membrane-bounded vesicle lumen	5	0.004091008
Vesicle lumen	5	0.004231613
Cell surface	12	0.007514831
Extracellular matrix component	5	0.008200888
Endoplasmic reticulum lumen	6	0.008374408
Cytoplasmic vesicle membrane	9	0.011071797
Vesicle membrane	9	0.013237355
Endoplasmic reticulum membrane	13	0.020041363
Endocytic vesicle	6	0.022348251
Nuclear outer membrane-endoplasmic reticulum membrane network	13	0.023319554
Plasma membrane receptor complex	5	0.023339645
Apical part of cell	7	0.024562127
Apical plasma membrane	6	0.035395124

**Table 2:** GO-MF analysis of HIN-AC sequence.

GO-MF term	Count	*P* value
Calcium ion binding	14	9.42*E* − 05
Protein complex binding	13	6.76*E* − 04
Glycosaminoglycan binding	7	8.21*E* − 04
Heparin binding	6	0.001594462
Carbohydrate derivative binding	23	0.002484656
Lipid transporter activity	5	0.002945673
Growth factor binding	5	0.004747338
Lipid binding	10	0.007765177
Sulfur compound binding	6	0.007899881
Macromolecular complex binding	15	0.008290491
Carboxylic acid binding	5	0.011207409
Transition metal ion binding	15	0.01677257
Metal ion binding	32	0.020098223
Cation binding	32	0.023441654
Ion binding	33	0.023514545
Protein dimerization activity	12	0.041811739
Receptor binding	14	0.043211111
Serine-type endopeptidase activity	5	0.046427575

**Table 3:** GO-BP analysis of HIN-AC sequence.

GO-BP term	Count	*P* value
Defense response to fungus	8	1.42*E* − 09
Response to fungus	8	1.34*E* − 08
Defense response to other organisms	15	3.91*E* − 07
Cell proliferation	28	1.01*E* − 06
Defense response to bacterium	11	1.70*E* − 06
Response to external stimulus	29	2.49*E* − 06
Positive regulation of cell proliferation	18	2.53*E* − 06
Response to biotic stimulus	18	3.69*E* − 06
Response to organic substance	34	5.47*E* − 06
Leukocyte migration	12	5.67*E* − 06
Regulation of cell proliferation	24	5.75*E* − 06
Response to external biotic stimulus	17	8.32*E* − 06
Response to other organisms	17	8.32*E* − 06
Enzyme linked receptor protein signaling pathway	18	1.19*E* − 05
Extracellular matrix organization	11	1.27*E* − 05
Extracellular structure organization	11	1.31*E* − 05
Positive regulation of response to external stimulus	10	1.39*E* − 05
Cell migration	20	1.49*E* − 05
Cellular response to endogenous stimulus	20	1.51*E* − 05
Response to endogenous stimulus	23	1.88*E* − 05

## Data Availability

The mass spectrometry proteomics data have been deposited in the ProteomeXchange Consortium (https://proteomecentral.proteomexchange.org) via the iProX partner repository with the dataset identifier: PXD023899.

## Supplementary Materials

Supplementary Materials Figure S1: workflow of the study. Figure S2: P3H1 is a risk factor for OS and DFS in the TCGA CRC dataset. Table S1: clinical and pathological characteristics of the patients. Table S2: top 5 hub proteins of MCODE 3 in different methods of CytoHubba plugin.
