# Supplementary material for: Exploration of the Key Proteins of High-Grade Intraepithelial Neoplasia to Adenocarcinoma Sequence Using In-Depth Quantitative Proteomics Analysis
Source: J Oncol. 2021 Nov 29;2021:5538756. doi: 10.1155/2021/5538756 (PMC8648452; doi:10.1155/2021/5538756)
Supplement: Supplementary Materials — Figure S1: workflow of the study. Figure S2: P3H1 is a risk factor for OS and DFS in the TCGA CRC dataset. Table S1: clinical and pathological characteristics of the patients. Table S2: top 5 hub proteins of MCODE 3 in different methods of CytoHubba plugin. [file 5538756.f1.docx]

**Figure S1** **Workflow of the study**


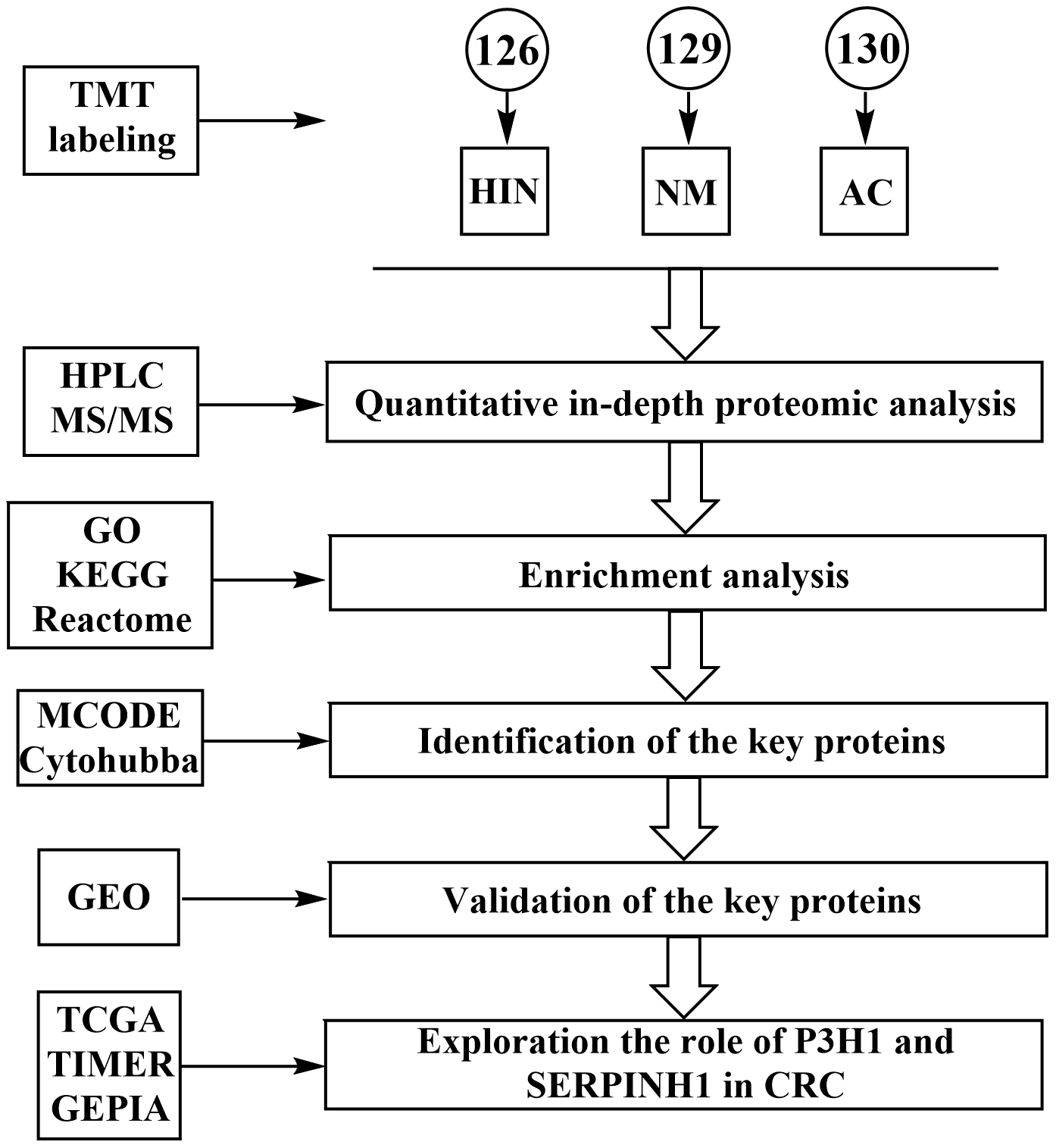


**Figure S2** **P3H1 is a risk factor for OS and DFS in the TCGA CRC dataset**

The OS of the high P3H1 expression group are significantly poorer than that of the low expression group (*P* =0.03, left). The DFS of the high P3H1 expression group are significantly poorer than that of the low expression group (*P* =0.04, right)
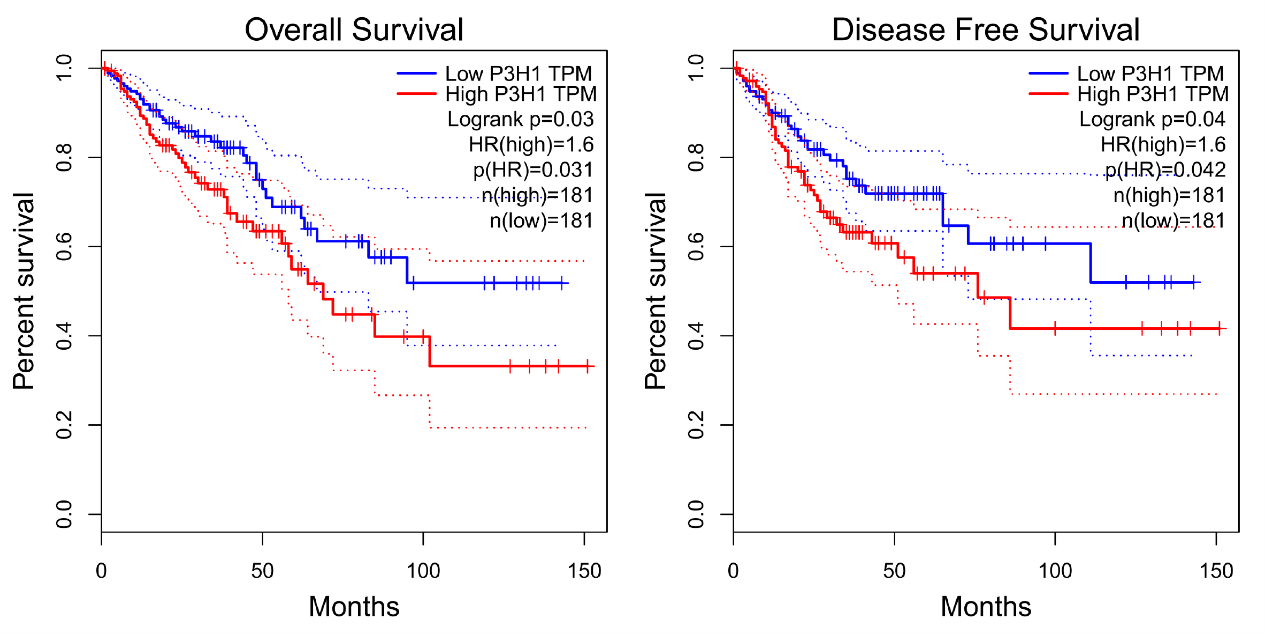


**Table S1 Clinical and pathological characteristics of the patients**

| **Patient** | **Sex** | **Age** | **Pathology** | **Location** | **Stage** | **Differentiation** |
| --- | --- | --- | --- | --- | --- | --- |
| 1 | M | 57 | NM | Asc | - | - |
| 2 | F | 50 | NM | Asc | - | - |
| 3 | F | 68 | NM | R | - | - |
| 4 | M | 59 | NM | Asc | - | - |
| 5 | M | 73 | NM | R | - | - |
| 6 | F | 54 | NM | Sc | - | - |
| 7 | F | 68 | NM | Sc | - | - |
| 8 | M | 80 | NM | Asc | - | - |
| 9 | M | 74 | HIN | R | - | - |
| 10 | F | 56 | HIN | RSc | - | - |
| 11 | M | 53 | HIN | Sc | - | - |
| 12 | F | 65 | HIN | Asc | - | - |
| 13 | F | 75 | HIN | Hf | - | - |
| 14 | M | 67 | HIN | Hf | - | - |
| 15 | F | 36 | HIN | R | - | - |
| 16 | M | 56 | HIN | Sf | - | - |
| 1 | M | 57 | AC | Ce | T3N0M0 | Moderate |
| 2 | F | 50 | AC | Ce | T3N0M0 | Moderate |
| 3 | F | 68 | AC | R | T2N0M0 | Well-Moderate |
| 4 | M | 59 | AC | Ce | T3N1bM0 | Well-Moderate |
| 5 | M | 73 | AC | R | T2N2aM0 | Moderate |
| 6 | F | 54 | AC | Sc | T3N0M0 | Moderate |
| 7 | F | 68 | AC | Sc | T3N2bM0 | Moderate |
| 8 | M | 80 | AC | Asc | T3N0M0 | Moderate |

Abbreviations: M, male; F, female; NM, normal mucosa; HIN, high-grade intraepithelial neoplasia; AC, adenocarcinoma; Ce, Cecum; Asc, ascending colon; Hf, hepatic flexture; Sf, splenic flexture; Sc, sigmoid colon; RSc, rectosigmoid colon; R, rectum.

**Table S2 Top 5 hub proteins of MCODE 3 in different methods of Cytohubba plugin**

| **MCC** | **DMNC** | **MNC** | **Clustering coefficient** |
| --- | --- | --- | --- |
| PLOD2 | PLOD2 | PLOD2 | PLOD1 |
| CRTAP | CRTAP | CRTAP | MNDA |
| FKBP10 | FKBP10 | FKBP10 | NCF4 |
| SERPINH1 | SERPINH1 | SERPINH1 | SERPINH1 |
| P3H1 | P3H1 | P3H1 | P3H1 |
